# Pyramiding of Four Broad Spectrum Bacterial Blight Resistance Genes in Cross Breeds of Basmati Rice

**DOI:** 10.3390/plants12010046

**Published:** 2022-12-22

**Authors:** Irfan Ullah, Hamid Ali, Tariq Mahmood, Mudassar Nawaz Khan, Muhammad Haris, Hussain Shah, Adil Mihoub, Aftab Jamal, Muhammad Farhan Saeed, Roberto Mancinelli, Emanuele Radicetti

**Affiliations:** 1 Department of Biotechnology and Genetic Engineering, Hazara University Mansehra, Mansehra 21300, Pakistan; 2 Department of Agriculture, Hazara University Mansehra, Mansehra 21300, Pakistan; 3 Plant Sciences Division, Pakistan Agricultural Research Council Islamabad, Islamabad 45500, Pakistan; 4 Center for Scientific and Technical Research on Arid Regions, Biophysical Environment Station, Toug-gourt 30240, Algeria; 5 Department of Soil and Environmental Sciences, Faculty of Crop Production Sciences, The University of Agriculture, Peshawar 25130, Pakistan; 6 Department of Environmental Sciences, Vehari-Campus, COMSATS University Islamabad, Vehari 61100, Pakistan; 7 Department of Agricultural and Forestry Sciences (DAFNE), University of Tuscia, 01100 Viterbo, Italy; 8 Department of Chemical, Pharmaceutical and Agricultural Sciences (DOCPAS), University of Ferrara, 44121 Ferrara, Italy

**Keywords:** *Oryza sativa*, pyramiding, bacterial blight, recombinant inbred lines, Basmati-385, response

## Abstract

Pyramiding of major resistance (*R*) genes through marker-assisted selection (MAS) is a useful way to attain durable and broad-spectrum resistance against *Xanthomonas oryzae* pv. *oryzae* pathogen, the causal agent of bacterial blight (BB) disease in rice (*Oryza sativa* L.). The present study was designed to pyramid four broad spectrum BB-*R* genes (*Xa*4, *xa*5, *xa*13 and *Xa*21) in the background of Basmati-385, an indica rice cultivar with much sought-after qualitative and quantitative grain traits. The cultivar, however, is susceptible to BB and was therefore, crossed with IRBB59 which possesses *R* genes *xa*5, *xa*13 and *Xa*21, to attain broad and durable resistance. A total of 19 F_1_ plants were obtained, some of which were backcrossed with Basmati-385 and large number of BC_1_F_1_ plants were obtained. In BC_1_F_2_ generation, 31 phenotypically superior genotypes having morphological features of Basmati-385, were selected and advanced up to BC_1_F_6_ population. Sequence-tagged site (STS)-based MAS was carried out and phenotypic selection was made in each successive generation. In BC_1_F_6_ population, potentially homozygous recombinant inbred lines (RILs) from each line were selected and evaluated on the bases of STS evaluation and resistance to local *Xanthomonas oryzae* pv. *oryzae* (*Xoo*) isolates. Line 23 was found pyramided with all four BB-*R* genes i.e., *Xa*4, *xa*5, *xa*13 and *Xa*21. Five genotypes including line 8, line 16, line 21, line 27 and line 28 were identified as pyramided with three *R* genes, *Xa*4, *xa*5 and *xa*13. Pathological study showed that rice lines pyramided with quadruplet or triplet *R* genes showed the highest level of resistance compared to doublet or singlet *R* genes. Thus, line 23 with quadruplet, and lines 8, 16, 21, 27, and 28 with triplet *R* genes, are recommended for replicated yield and resistance trials before release as new rice varieties. Further, traditional breeding coupled with MAS, is a solid way to attain highly effective BB-resistant rice lines with no yield cost.

## 1. Introduction

Rice (*Oryza sativa* L.) as one of the most important cereal crops and major source of nutrition is feeding about 2.5 billion people around the world. Asian countries are the main producers and consumers of rice, with China and India contributing to more than half of the global rice production. *O. sativa* and *O. glaberrima* (Steud.) [1] are two cultivated species of rice grown in Asia and Africa, respectively [2]. Currently, about 500 million tonnes (Mt) of milled rice is produced from about 160 million hectares (Mha) of global land [3]. Rice production needs to be increased by 42% from the current level to feed growing human population by 2050 [4]. The positive trend in rice production is, in principle, maintained by cultivating high yielding semi-dwarf varieties [5].

In Pakistan, rice is the third largest crop after wheat and cotton, and the second largest export commodity. Pakistan produced 7.41 million tonnes of rice in 2020 [6]. Its featured rice variety, Basmati, is a high valued group of rice cultivars famous for its unique organoleptic and cooking properties. The variety has a distinctive long grain which elongates to nearly double upon cooking, coupled with its signature aroma both as raw and cooked forms [7]. The variety, however, is highly susceptible to bacterial blight (BB) disease, compromising its yield and quality, and compelling farmers to resort to nonaromatic rice varieties [8]. Basmati-385, a long grained, early maturing, highly aromatic, high yielding and major export of Pakistan, is also susceptible to BB [9,10,11,12].

More than 40% of the world’s rice is lost annually due to many biotic stresses such as pests, insects, weeds and pathogens [2]. BB, caused by *Xanthomonas oryzae* pv. *oryzae* (*Xoo*) [13], is one of the most destructive diseases of rice. The disease causes 20–40% yield loss at tillering stage and, overall, 50–80% yield loss in case of severity [14,15]. BB epidemics have occurred several times in Pakistan and has severely affected many cultivars including highly aromatic and valuable Basmati genotypes [16]. The latest survey showed Punjab, the largest rice growing province of the country had the highest percentage (30–55%) of the disease incidence in its Basmati producing “Kaller” belt including Muridke, Narang, and adjoining areas. Following Punjab, Khyber Pukhtunkhwa province had 37% of the disease incidence, while Sindh province had the least incidence of 10–13% [17]. The disease can be managed in several ways including chemical methods and cultural practices. Nevertheless, breeding efforts to produce new Basmati varieties resistant to the disease vis-a-vis retaining its qualitative and quantitative properties, have increased [18,19,20,21].

To date, 45 genes conferring resistance against BB, have been identified [22,23,24]. Among these resistance genes, 17 (*xa*5, *xa*8, *xa*13, *xa*15, *xa*19, *xa*20, *xa*24, *xa*25, *xa*26, *xa*28, *xa*31, *xa*32, *xa*33, *xa*34, *xa*41, *xa*42 and *xa*44) are recessive, while the remaining 28 are dominant [24,25,26,27]. Nine of the resistance genes, *Xa*1, *Xa*3/*xa*26, *xa*5, *Xa*10, *xa*13, *Xa*21, *Xa*23, *xa*25 and *Xa*27, have been cloned, while twelve genes, *Xa*2, *Xa*4, *Xa*7, *Xa*22, *Xa*30, *xa*31, *xa*33, *xa*34, *Xa*38, *Xa*39, *Xa*40 and *Xa*42, have been physically mapped [24,25,26,27,28,29,30,31,32,33,34,35]. Closely linked functional markers of these genes are available for marker-assisted introgression into desired cultivars [36]. The Intentional Rice Research Institute (IRRI) developed several BB-resistant cultivars, named near-isogenic lines or IRBB series. For example, IRBB5, IRBB7, IRBB21, and IRBB59, carrying the *xa*5, *Xa*7, *xa*13, and *Xa*21 genes, respectively, for BB resistance. These lines have become popular donor parents in rice improvement programs for BB resistance worldwide. BB-*R* genes, *Xa*4, *xa*5, *Xa*7, *xa*13 and *Xa*21, are widely being used in resistance breeding programs [37,38,39]. A single BB-*R* gene, however, cannot provide durable resistance against the prevalent pathotypes of *Xoo,* and thereby, led to the combination of genes for broad and durable resistance [40]. Pyramiding of multiple BB-*R* genes is an effective approach to cope with *Xoo* strains from diverse rice growing areas in the world [41]. Pyramiding of *xa*13+ *Xa*21 [42], *Xa*4 + *xa*5 + *Xa*21 [43] and *Xa*4 + *xa*5 + *xa*13 + *Xa*21 [44], has been reported to provide durable BB resistance as an effective repertoire of *R* genes compared to single *R* genes [15].

Conventional breeding-based pyramiding for BB resistance without the use of molecular markers does not provide complete picture of the phenomenon. Furthermore, genes with similar reactions to two or more races of the *Xoo* pathogen are tedious to be identified and transferred through only conventional methods, greatly necessitating the utilization of MAS approach. The availability of molecular markers tightly linked with each of the *R* genes makes the identification of plants with two or more genes possible [45]. In Pakistan, none of the approved Basmati varieties provides resistance to BB disease, indicating that the Basmati germplasm lacks effective *R* genes in its genetic pool [46]. It is thus possible to develop new Basmati rice cultivars with high-level and long-lasting resistance to BB by pyramiding several *R* genes into a single cultivar. The present study was, therefore, proposed to pyramid BB-R genes *Xa4*, *xa5*, *xa13* and *Xa21*, into rice cultivar Basmati-385.

## 2. Results

### 2.1. PCR Analysis

**Figure 1:**
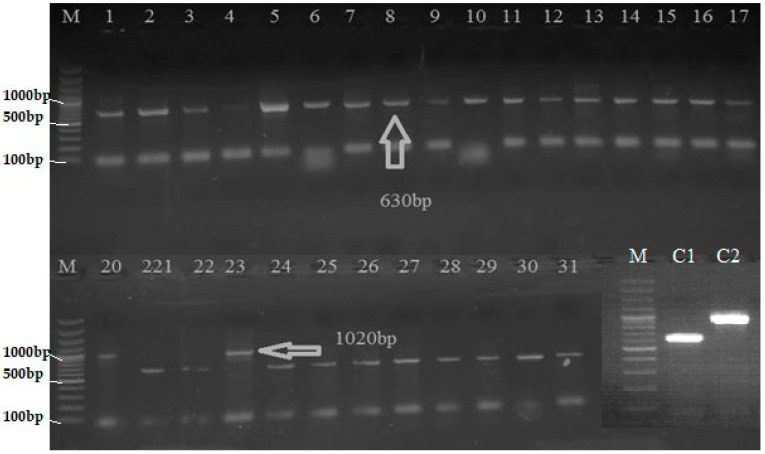
PCR analysis of RILs of rice for the presence of *Xa21* gene (Arrow showing 1020 bp bands of *Xa*-21 gene). M = 100 bp DNA ladder, C1 = Basmati-385, C2 = IRBB59, line 1–31 = Selected genotypes.

**Figure 2:**
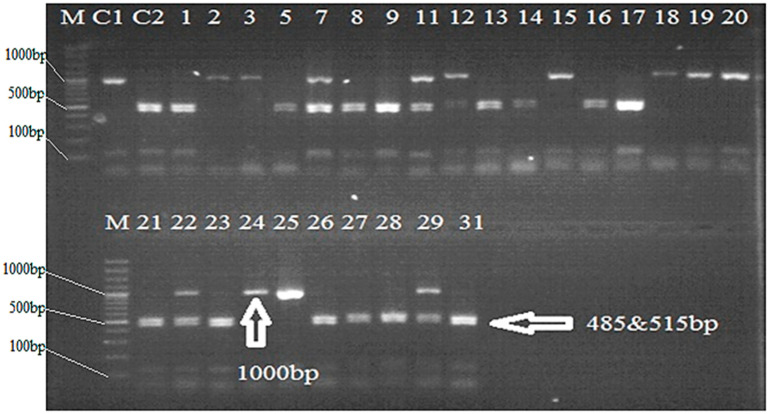
PCR analysis of RILs of rice for the presence of *xa13* gene (Arrow showing 485 and 515 bp bands of *xa*13 gene). M = 100 bp DNA ladder, C1 = Basmati-385, C2 = IRBB59, line 1–31 = Selected genotypes.

**Figure 3:**
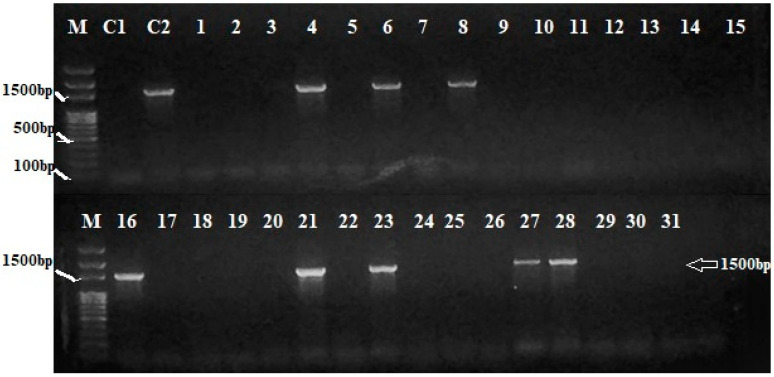
PCR analysis of RILs of rice for the presence of *xa5* gene (Arrow showing 1500 bp bands linked to *xa*5 gene). M = 100 bp DNA ladder, C1 = Basmati-385, C2 = IRBB59, lines 1–31 = Selected genotypes.

**Figure 4:**
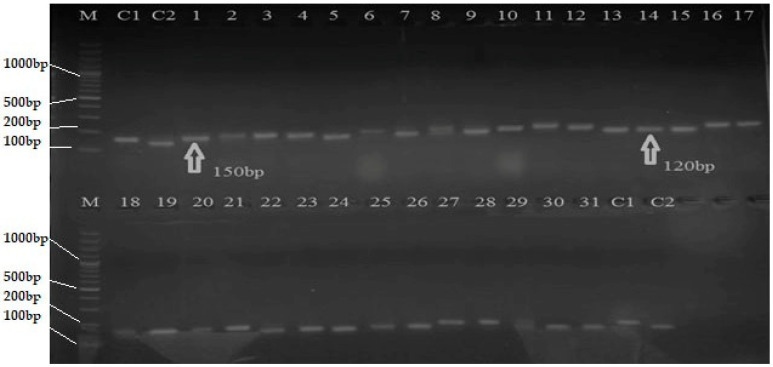
PCR analysis of RILs of rice for the presence of *Xa*4 gene (Arrow showing 150 bp bands linked to *Xa*4 gene). M = 100 bp DNA ladder, C1 = Basmati-385, C2 = IRBB59, line 1–31 = Selected genotypes.

An amplicon of 1020 bp was observed in IRBB59, when a pair of *Xa21*-specific primer (pTA248) was used in PCR analysis, thus confirming the presence of *Xa21* gene. Among the recombinant inbred lines (RILs), two lines, 20 and 23, showed 1020 bp bands of *Xa21* gene, which was lacking in all other genotypes (Table 1; Figure 1). Similarly, sequence-tagged site (STS) primer RG136 was used for amplification of *xa*13-linked DNA fragment. A monomorphic band of 1000 bp was amplified in all the genotypes. To achieve polymorphism, the 1000 bp monomorphic band was restricted with *Hinf* I enzyme in all RILs. In total, 19 out of 31 RILs gave two bands of 485 and 515 bp for resistant *xa*13 allele, while 12 genotypes gave just a single band of 1000 bp of susceptible *Xa13* (Table 1; Figure 2). An amplicon of 1500 bp was observed in IRBB59, when a pair of *xa*5 specific primers (RZ207) was used in PCR analysis, thus confirming the presence of *xa*5 gene. Out of 31 RILs, only 8 genotypes showed 1500 bp bands, indicating the presence of *xa*5 gene (Table 1; Figure 3). An amplicon of 150 bp was observed in Basmati-385 when *Xa*4-linked primers (MP1, MP2) were used. Out of 31 RILs, 18 genotypes showed the presence of 150 bp band and thus possessed *Xa*4 gene. IRBB-59 and the remaining 13 genotypes, on the other hand, were observed with 120 bp band, thus lacking in *Xa*4 gene (Table 1; Figure 4).

### 2.2. Identification of Pyramided Lines in BC_1_F_6_ Population

**Figure 5:**
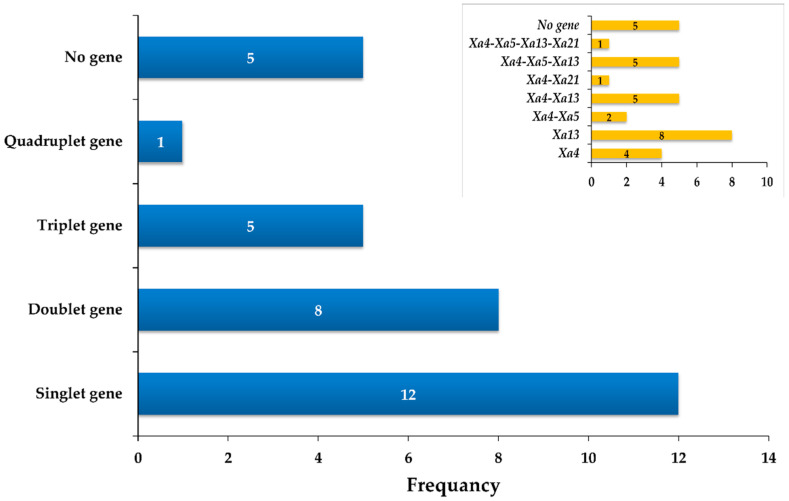
Combination and frequency of *Xa* genes in RILs of rice.

Line-23 was identified to possess all the four BB-R genes (*Xa*4, *xa*5, *xa*13 and *Xa*21) while line-8, line-16, line-21, line-27 and line-28 possessed three *R* genes among the advanced BC_1_F_6_ population developed in the present study (Figure 5).

### 2.3. Responses of RILs to BB Isolates

Three local isolates of BB were used to evaluate the responses of selected genotypes. Analysis of variance showed significant differences among the genotypes for mean lesion length developed by isolates *Xoo*1, *Xoo*2, and *Xoo*3. Lesion size of RILs on inoculation with *Xoo*1 ranged from 1.3 to 60.1% with mean value of 24.1%, followed by 1-58% with mean value of 23.7% when inoculated *Xoo*2. Inoculation of *Xoo*3 gave 2-66.5% of lesion size with overall mean of 25.8% (Table 2).

**Figure 6:**
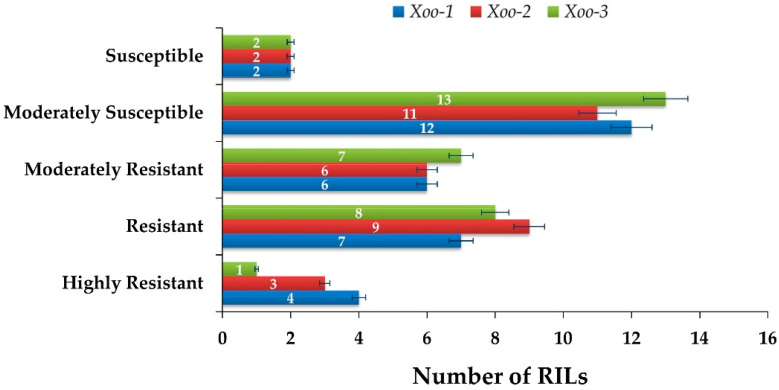
Response of RILs plants to BB isolates.

**Figure 7:**
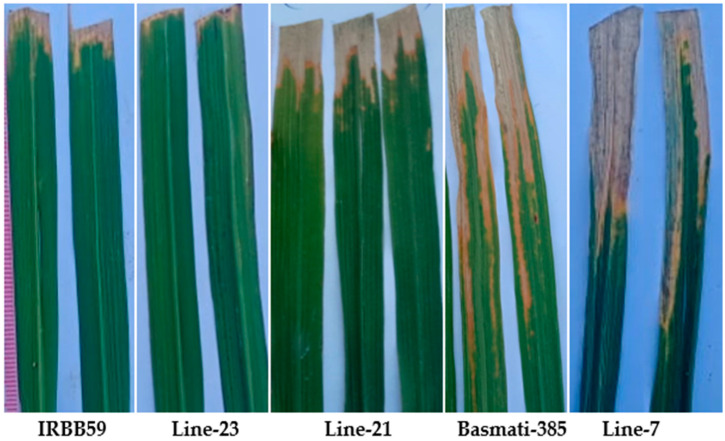
Lesion developed by *Xoo* isolates on advanced RILs of rice.

**Figure 8:**
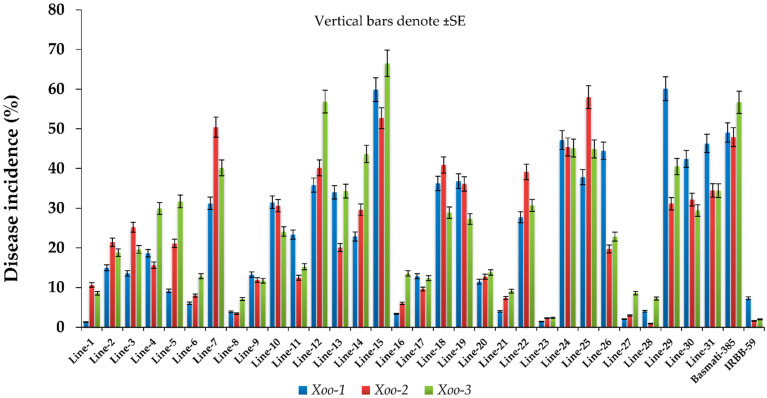
Bar graph showing diseases incidence (%). Vertical bars denote ± SE.

Out of 31 RILs, 18 genotypes showed resistant reactions to *Xoo1*, while 15 genotypes showed susceptible reactions. Among the former, lines 1, 16, 23 and 27 were highly resistant (HR), lines 5, 6, 8, 17, 20, 21 and 28 were resistant (R) and lines 2, 3, 4, 9, 11 and 14 were moderately resistant (MR). Among the latter, lines 7, 10, 12, 13, 18, 19, 22, 24, 25, 26, 30, 31 and Basmati-385 were moderately susceptible (MS), while lines 15 and 29 were susceptible (S). Similarly, 19 genotypes showed resistant reactions, while 14 genotypes showed susceptible reactions to *Xoo2*. Among the former, lines 23, 27, 28 and IRBB-59 showed HR, lines 1, 6, 8, 9, 11, 16, 17, 20 and 21 showed R and lines 2, 3, 4, 5, 13 and 26 showed MR reactions. Among the later, lines 7, 10, 12, 14, 18, 19, 22, 24, 29, 30, 31 and Basmati-385 showed MS reaction, while lines 15 and 25 showed S reaction to *Xoo2*. Similarly, 17 and 16 genotypes showed R and S reactions, respectively, to *Xoo3*. Among the former, line 23 and IRBB-59 showed HR, lines 1, 6, 8, 9, 17, 21, 27 and 28 R, while lines 2, 3, 10, 11, 16, 20 and 26 MR responses. Among the later, lines 4, 5, 7, 13, 14, 18, 19, 22, 24, 25, 29, 30 and 31 showed MS response, while line 12 and 15 and Basmati-385 showed S response to *Xoo3* (Table 2; Figure 6, Figure 7 and Figure 8).

**Figure 9:**
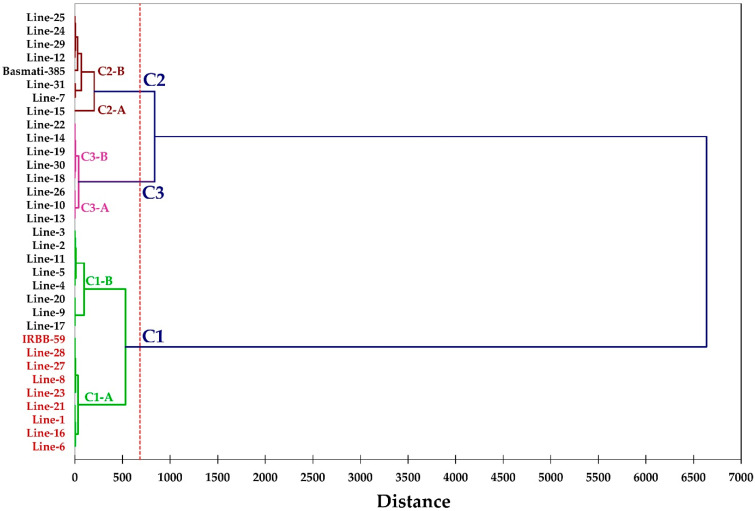
Phylogenetic tree constructed on the basis of percent diseases incidence.

A phylogenetic tree was constructed based on percent diseases incidence. The tree was divided into three main clades. Clade 1 was further subdivided into two sub clades, C1A and C1B. Clade C1A consisted of 9 genotypes, including lines 6, 16, 21, 1, 8, 27, 28, 23 and IRBB-59 and was the most important clade consisting of all resistant genotypes. Both clades 2 and 3 comprised of 8 genotypes, each of which were further subdivided into two sub clades. The genotypes present in these clades were moderately susceptible to *Xoo* isolates (Table 2; Figure 9).

## 3. Discussion

Basmati rice is praised all over the world for its traits of unique taste, aroma and grain length. Its characteristic morpho-physiological ideotypes, however, lacks resistance against the BB disease in almost all tropical rice growing regions of the world [47], considerably slashing the net rice productivity. The approach of ‘defect elimination’ of crop ideotype was used in this study to introgress BB resistance genes from IRBB59 to the susceptible Basmati-385 variety [48]. Introgression of *R* genes in Basmati varieties aided by molecular markers has been well reviewed in BB management programs [49]. Out of 45 BB resistance (R) genes identified [22,23], *R* genes *Xa*4, *xa*5, *Xa*7, *xa*13 and *Xa*21, have been individually incorporated into rice cultivars. However, the single gene-associated resistance is prone to break down and has been overcome by new strains of the pathogen. MAS-based pyramiding of major *R* genes into a single genotype seems to be a resource-feasible approach to attain durable and broad-spectrum resistance [20]. The approach was adopted in the current study to pyramid quadruplet BB-*R* genes in Basmati-385 rice background.

The quadruplet *R* genes have been exclusively and successfully pyramided in different rice varieties or their backgrounds. Resistance was achieved in rice variety Tapaswini for lowland ecology by introgressing *Xa4*, *xa5*, *xa13*, and *Xa21* genes [50,51]. The disease incidence of Putra-1 was significantly lowered to 6.35% from 53% in the introgressed F_1_ lines when pyramided with quadruplet *Xa4*, *xa5*, *xa13*, and *Xa21* from the donor IRBB60 [15]. Similarly, the Indian susceptible varieties Mahsuri, PRR78, and KMR3 were bred for resistance by pyramiding *Xa*4, *xa*5, *xa*13 and *Xa*21 *R* genes, resulting into 1–3 cm infection in the resistant lines compared to lesion lengths of 22.6 cm, 18.8 cm and 18.2 cm in the susceptible parents [52]. The resistance response of the CNYBB4R03 line pyramided with *Xa*4, *xa*5, *xa*13 and *Xa*21 genes was 0.43 cm compared to 6.75–12.56 cm in susceptible varieties, TNG82, TCS10, TN1 and IR24 [38]. In the current study, RIL 23 pyramided with *Xa*4, *xa*5, *xa*13 and *Xa*21 genes showed highest level of resistance with just 1.4–2.4% disease lesion length compared to Basmati-385 in response to local Himalayan *Xoo* strains.

Similar to quadruplets, combination of triplet *R* genes also showed significant BB-resistance in different susceptible rice cultivars [45,53]. The triplet set of *R* genes, *Xa*4, *xa*5 and *Xa*21, has been exclusively used in different rice breeding for BB resistance programs. The triplet-genes-transformed CSR-30 rice cultivar, for example, exhibited an incompatible mean lesion length of 0.4 cm, near to the mean lesion length of 0.5 cm of donor IRBB-60 against *Xoo*. Similarly, the high-yielding deep-water rice variety, Jalmagna and PKBB 15–116 lines showed reduced lesions of 1.4–2.9 cm and 4% compared to 9.4–12.8 cm and 60% in the respective controls when pyramided with *Xa4, xa5*, and *Xa21* triplet genes [41,46]. Pyramided with *Xa*4, *xa*5, and *Xa*21 from donor line IRBB57, the Korean elite japonica variety Mangeumbyeo, showed a drastic regression of <0.3 cm lesion length, near to the <0.5 cm lesion length of donor IRBB-57, compared to 9-18 cm lesion length when challenged with 18 *Xoo* isolates. The NILs introgressed with either of individual *R* genes, *Xa4, xa5* or *Xa21* were, however, exhibited S, MR and R reactions, respectively, to the 18 isolates in question [54]. In the current results, 6 RILs having three genes *Xa*4, *xa*5 and *xa*13, showed high resistance reaction compared to RILs with single or double genes, suggesting the additive nature of the resistance.

The mean lesion length on the doublet *R* genes combinations: *Xa*21 and *xa*13, *Xa*21 and *xa*5, and *xa*5 and *xa*13 was 3.1–3.9 cm, 3.5–4.8 cm, and 4.9–5.7, respectively [41]. *Xa21* and *xa*5, individually, showed resistance with the mean lesion lengths of 1.2 cm and 1.1 cm, respectively, though *xa13*-harboring genotype was moderately resistant with of 4.8 cm lesion length when challenged with *Xoo* [53]. Rashid et al., [55] studied the response of different lines to 118 local *Xoo* isolates and found the lines with single *R* genes did not show much resistance. There were, however, pyramided lines with *xa*5 and *xa*13 or *xa*13 and *Xa*21 genes combinations, showing the highest amount of resistance frequency (100%). Similarly, Yugander et al., [56] observed IRBB lines with single *R* genes were susceptible to 73.2–97.2% of the isolates. Current study showed that the quadruplet- and triplet-genes-introgressed lines were the best lines which showed resistance against the selected Himalayan *Xoo* strains, corroborating the cumulative impact of multiple *R* genes.

The epistatic effect of the dominant *R* genes on plant morphology and physiology, and quantitatively indistinguishable impact between different *R* genes on two or more races of *Xoo*, make the identification of and differentiation between different *R* genes a tough task. Molecular markers tightly linked with each of the *R* genes make the identification of plants with two or more *R* genes possible [45]. Several genes for resistance to BB have been tagged with molecular markers [57]. These include restriction fragment length polymorphism, randomly amplified polymorphic DNA markers and STS markers to validate the presence of introgressed *R* genes in lines of interest [58]. It is possible to develop closely linked molecular markers for each of the *R* genes within a plant [45,59]. The potential of STS markers was, therefore, assessed to identify rice lines with multiple BB-*R* genes in the current study. These RILs developed were resistant to the selected strains collected from the foothills of Himalayas in Mansehra district, which otherwise, were virulent to the parent Basmati 385. The local farmers of the district can grow Basmati-like RILs in the foothills of Himalaya, unlike the parent Basmati-385 which is generally grown in the Punjab province of Pakistan, without any significant BB disease incidence. Further, such RILs can be tested in the mainstream rice growing regions of Pakistani Punjab and its adjoining Indian Punjab, to evaluate their qualitative and quantitative traits before their adoption in these mainstream regions.

## 4. Materials and Methods

### 4.1. Pyramiding BB-R Genes into Basmati-385

**Figure 10:**
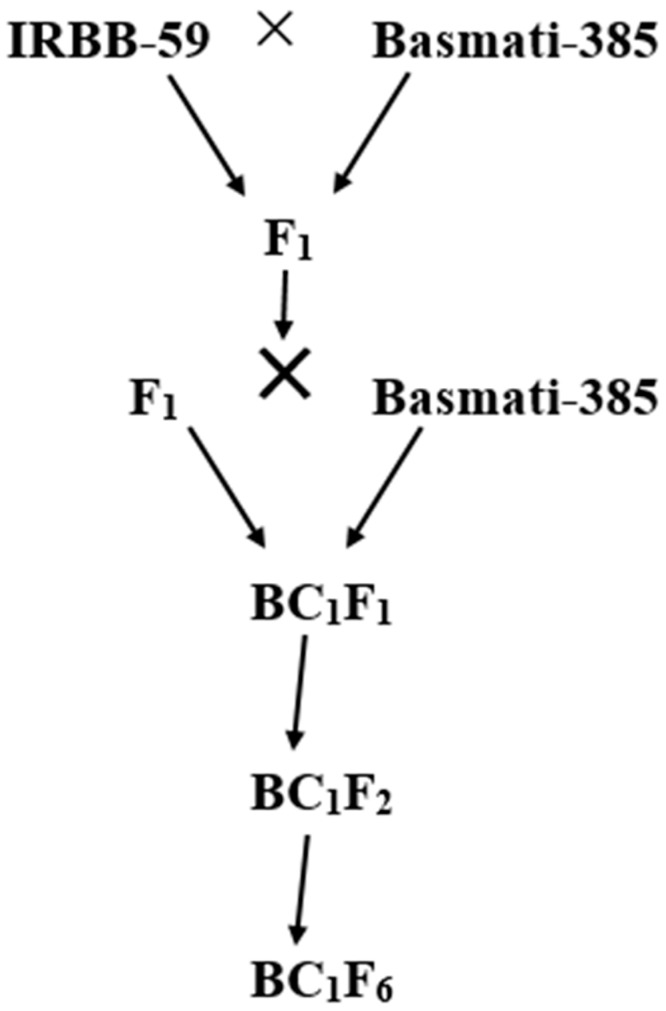
Breeding scheme for developing pyramided lines.

Basmati-385 was crossed with IRBB-59, in which the former contributes single BB-*R* gene *Xa4*, while the later contributes triple BB-*R* genes, *Xa21, xa5* and *xa13* (Figure 10).

The breeding program was started in rice growing season 2014. A large number of immature spikelets of Basmati-385 were emasculated early in the morning and shed with sufficient pollens of IRBB59 in the afternoon. The pollinated panicles were covered with crossing bags. The hybrid seeds were harvested 30 days after pollination. A total of 19 hybrid seeds were obtained and germinated in sterile petri plates to grow F_1_ hybrids. Some of the F_1_ hybrids were back crossed with Basmati-385 and thus BC_1_F_1_ population was obtained and selfed to produce a large number of BC_1_F_2_ population. Phenotypically, superior plants were advanced up to BC_1_F_6_ population. Finally, 31 potentially homozygous recombinant inbred lines (RILs) were selected and evaluated on the bases of molecular markers and their resistance to local *Xoo* isolates.

### 4.2. Extraction of Genomic DNA

The genomic (g) DNA was extracted using CTAB method [20]. Fresh leaves at early seedling stages were collected in Eppendorf tubes and put immediately in liquid nitrogen. Crushed with a glass rod and 700 µL of heated (60 °C) 2× CTAB buffer (50 mM Tris-HCl, pH 8.0, 25 mM EDTA, 300 mM NaCl and 2% CTAB) was added to each sample. The samples were then incubated at 56 °C overnight and mixed again with the help of a glass rod. Followed by addition of 700 µL Chloroform: Isoamyl alcohol (24:1) solution and kept at room temperature for 30 min. The samples were then centrifuged at 9000 rpm for 20 min and a clear supernatant (500 µL) was transferred to a newly labeled Eppendorf tubes. Then 500µL ice cold isopropanol and 40 µL sodium acetate were added to it and incubated at −20 °C for at least 1 h or overnight. The samples were centrifuged at 9000 rpm for 20 min to make DNA pellet. The supernatant was discarded, and the pellet was washed with 70% ethanol and dried at room temperature. Then, 40 µL TE buffer was added to each sample. For RNA degradation 1µL RNAse was added to each tube and incubated at 37 °C for one hour. The quality and quantity of DNA was checked on 1% agarose gel stained with ethidium bromide. The concentration of DNA was adjusted from 20 to 50 ng/µL by using double distilled water and stored at 4 °C for further use.

### 4.3. PCR Amplification of Bacterial Blight R Genes

PCR reactions were carried out in 50 µL reaction volumes having 1–2 µL genomic DNA, 1 µL each of forward and reverse STS primers. Amplification was carried out in DNA thermal Cycler (Applied Bio System), at 94 °C for 6 min as initial denaturation, 36 cycles at 94 °C for 1 min, 55 °C for 1 min, and 72 °C for 2 min. Final extension was carried out at 72 °C for 7 min. PCR products were run on 2% agarose gel in TAE buffer. For identification of *xa*13 gene, initially 5µL PCR product was used in gel electrophoresis. The remaining PCR product was used for restriction digests. The reaction mixture for restriction of PCR amplicon of *xa*13 gene consisted of 3.2 µL sterile distilled water, 1.5 µL restriction buffer (tango buffer), 0.3 µL restriction enzyme *Hinf1*(10 U/µL) and 15 µL of PCR product. Incubation of the reaction mixture varied from 4 h to overnight at 37 °C. The resultant fragments were separated by gel electrophoresis on 1.5% agarose gel. The gel was stained with ethidium bromide (10 ug/mL) and observed under UV light (Table 3).

### 4.4. Xanthomonas oryzae pv. oryzae (Xoo) Strain Isolation

Rice leaves showing clear BB symptoms were collected from various rice fields at district Mansehra and kept in refrigerator. For isolation of bacterial strains, the samples were washed with sterilized distilled water and air dried in laminar flow hood. Four Eppendorf tubes were filled with sterilized water, while one Eppendorf tube with 70% ethanol. Leaves were cut at points of fresh disease symptoms into 2–4 cm fragments and washed with 70% ethanol, followed by rinsing with sterile distil water 2–3 times and crushed in 1 mL sterilized water using sterilized blue tip in a fresh Eppendorf tube. The crushed material was incubated at 30 °C for one hour so that bacteria may ooze out into the water. After dipping the sterilized wire loop into the crushed material, bacteria were streaked on the petri dishes having nutrient agar media, and kept in incubator for 4 days at 28 °C. Pure cultures were derived from single round, smooth, golden yellow and mucous colonies and streaked in new petri dishes having nutrient agar media and incubated for 2–3 days at 28 °C.

### 4.5. Preparation of Inoculum from Pure Cultures

For preparation of inoculum 10–15 mL distilled water was taken in 30 mL tubes and mixed with a 2-days pure cultures of *Xoo*. Selected genotypes were inoculated at seedling stages using clip method as reported by [63]. Disease symptoms were observed on a daily basis till the 16th day and compared with Intentional Rice Research Institute (IRRI) standard scale as reference for diseases scoring (Table 4).

### 4.6. Data Analyses

Analysis of variance (ANOVA) was performed and Tukey test was used for comparison of means using statistical software Statistix 8.1. Cluster analysis was performed using Past software.

## 5. Conclusions

The present study conclusively proves RIL 23 as the only quadruplet-genes-introgressed line showing the best resistance against the selected Himalayan *Xoo* strains, followed by 5 triplet-genes-introgressed RILs consisting of 8, 16, 21, 27 and 28. Though having the high possibility of adoption in Northern Pakistan, country-wide multi-location trials for the RILs are needed to evaluate their resistance against extant of *Xoo* strains and yield performance before incorporating them in national rice breeding programs.

## Figures and Tables

**Table 1:** PCR analysis of RILs for bacterial blight R genes.

Genotypes	*Xa*-4	*xa*-5	*xa-*13	*Xa*-21
Line-1	+	-	+	-
Line-2	+	-	-	-
Line-3	+	-	-	-
Line-4	+	+	-	-
Line-5	-	-	+	-
Line-6	+	+	-	-
Line-7	-	-	+/-	-
Line-8	+	+	+	-
Line-9	+	-	+	-
Line-10	-	-	-	-
Line-11	+	-	+/-	-
Line-12	+	-	+/-	-
Line-13	-	-	+	-
Line-14	-	-	+	-
Line-15	-	-	-	-
Line-16	+	+	+	-
Line-17	+	-	+	-
Line-18	+	-	-	-
Line-19	+	-	-	-
Line-20	+	-	-	+
Line-21	+	+	+	-
Line-22	-	-	+/-	-
Line-23	+	+	+	+
Line-24	-	-	-	-
Line-25	-	-	-	-
Line-26	-	-	+	-
Line-27	+	+	+	-
Line-28	+	+	+	-
Line-29	-	-	+/-	-
Line-30	-	-	-	-
Line-31	-	-	+	-
Basmati-385	+	-	-	-
IRBB-59	-	+	+	+

“+” for presence and “-” for absence of *Xa* genes.

**Table 2:** Lesion percentage and responses of selected RILs of rice.

Genotypes	Lesion Length (%)			Reaction to Isolates		
	*Xoo*1	*Xoo*2	*Xoo*3	*Xoo*1	*Xoo*2	*Xoo*3
Line-1	1.3 ^J^	10.6 ^J–L^	8.5 ^K–M^	HR	R	R
Line-2	15 ^G–J^	21.4 ^G–K^	18.8 ^G–L^	MR	MR	MR
Line-3	13.6 ^G–J^	25.2 ^F–J^	19.6 ^G–L^	MR	MR	MR
Line-4	18.6 ^F–I^	15.6 ^I–L^	29.9 ^D–G^	MR	MR	MS
Line-5	9.1 ^H–J^	21.1 ^G–K^	31.7 ^C–G^	R	MR	MS
Line-6	6 ^IJ^	8 ^KL^	12.8 ^I–M^	R	R	R
Line-7	31.2 ^C–F^	50.4 ^A–C^	40.1 ^C–E^	MS	MS	MS
Line-8	3.9 ^IJ^	3.4 ^L^	7.1 ^LM^	R	R	R
Line-9	13.2 ^G–J^	11.9 ^J–L^	11.7 ^J–M^	MR	R	R
Line-10	31.5 ^C–F^	30.6 ^E–I^	24.1 ^F–J^	MS	MS	MR
Line-11	23.3 ^E–H^	12.5 ^J–L^	15.3 ^H–M^	MR	R	MR
Line-12	35.8 ^B–E^	40.1 ^B–F^	56.8 ^AB^	MS	MS	S
Line-13	34 ^B–F^	20.1 ^H–K^	34.3 ^C–F^	MS	MR	MS
Line-14	22.9 ^E–H^	29.6 ^E–I^	43.7 ^B–D^	MR	MS	MS
Line-15	59.8 ^A^	52.7 ^AB^	66.5 ^A^	S	S	S
Line-16	3.3 ^IJ^	6 ^KL^	13.6 ^I–M^	HR	R	MR
Line-17	12.8 ^G–J^	9.6 ^J–L^	12.4 ^J–M^	R	R	R
Line-18	36.2 ^B–E^	40.9 ^B–F^	28.8 ^E–H^	MS	MS	MS
Line-19	36.8 ^B–E^	36.1 ^C–G^	27.2 ^E–I^	MS	MS	MS
Line-20	11.5 ^H–J^	12.7 ^J–L^	13.8 ^I–M^	R	R	MR
Line-21	4 ^IJ^	7.4 ^KL^	9.1 ^K–M^	R	R	R
Line-22	27.7 ^D–G^	39.1 ^B–F^	30.7 ^C–G^	MS	MS	MS
Line-23	1.4 ^J^	2.3 ^L^	2.4 ^M^	HR	HR	HR
Line-24	47.2 ^A–C^	42.4 ^A–E^	45.1 ^BC^	MS	MS	MS
Line-25	37.8 ^B–E^	58 ^A^	44.9 ^BC^	MS	S	MS
Line-26	44.5 ^A–C^	19.7 ^H–K^	22.8 ^F–K^	MS	MR	MR
Line-27	2.1 ^J^	2.9 ^L^	8.6 ^K–M^	HR	HR	R
Line-28	4.1 ^IJ^	1 ^L^	7.2 ^LM^	R	HR	R
Line-29	60.1 ^A^	31.1 ^E–I^	40.5 ^C–E^	S	MS	MS
Line-30	42.5 ^B–D^	32.1 ^D–H^	29.4 ^D–H^	MS	MS	MS
Line-31	46.3 ^A–C^	34.5 ^D–H^	34.4 ^C–F^	MS	MS	MS
Basmati-385	49.1 ^AB^	47.9 ^A–D^	56.7 ^AB^	MS	MS	S
IRBB-59	7.3 ^H–J^	1.6 ^L^	2 ^M^	R	HR	HR
Range	60.1–1.3	58–1	66.5–2			
Grand mean	24.1	23.7	25.8			

The same superscript letters in the columns depict non-significant difference of values between the lines at 5% probability levels. HR = Highly Resistant, R = Resistant, MR = Moderately Resistant, MS = Moderately Susceptible, S = Susceptible.

**Table 3:** Sequence of primers used in this study.

Primer	Primer Sequence (5′ to 3′)	Linked Gene	Reference
MP 1, 2	(F) ATCGATCGATCTTCACGAGG	*Xa4*	[60]
	(R) TGCTATAAAAGGCATTCGGG		
RZ207	(F) GCCTCGAGCATCATCATCAG	*xa*5	[61]
	(R) ATCAACCTGCACTTGCCTGG		
RG136	(F) TCCCAGAAAGCTACTACAGC	*xa*13	[57]
	(R) GCAGACTCCAGTTTGACTTC		
pTA248	(F)AGACGCGGAAGGGTGGTTCC CGGA	*Xa*21	[62]
	(R) AGACCGGTAATCGAAAGATGAAA		

**Table 4:** Bacterial blight disease rating scale.

Groups	Lesion Percentage	Disease Rating Scale
HR (Highly Resistant)	0–3	1
R (Resistant)	4–12	3
MR (Moderately Resistant)	12–25	4
MS (Moderately Susceptible)	25–50	5
S (Susceptible)	51–87	7
HS (Highly Susceptible)	87–100	9

## Data Availability

Not applicable.

## References

[1] Linares O.F. (2002). African rice (*Oryza glaberrima*): History and future potential. Proc. Natl. Acad. Sci. USA.

[2] Noreen R., Khan S., Rabbani A., Kanwal A., Uzair B. (2020). Screening of different rice (*Oryza sativa* L.) varieties for genetic diversity and bacterial blight R gene. Pak. J. Bot..

[3] USDA (2020). World Agricultural Production. https://www.fas.usda.gov/data/world-agricultural-production.

[4] Ray D.K., Mueller N.D., West P.C., Foley J.A. (2013). Yield trends are insufficient to double global crop production by 2050. PLoS ONE.

[5] Gnanamanickam S.S. (2009). Rice and its importance to human life. Biological Control of Rice Diseases.

[6] Gul A., Xiumin W., Chandio A.A., Rehman A., Siyal S.A., Asare I. (2022). Tracking the effect of climatic and non-climatic elements on rice production in Pakistan using the ARDL approach. Environ. Sci. Pollut. Res..

[7] Woolfe M., Steele K. (2019). The Authenticity of Basmati Rice—A Case Study. DNA Techniques to Verify Food Authenticity.

[8] Ahmad M., Akhtar M., Anwar M. Basmati rice: Progress, issues and prospects for Pakistan. Proceedings of the International Seminar in Rice Crop.

[9] Bashir K., Khan N.M., Rasheed S., Salim M. (2007). Indica rice varietal development in Pakistan: An overview. Water Environ..

[10] Akram M. (2009). Aromatic rices of Pakistan—A review. Pak. J. Agric. Res..

[11] Saleem M.Y., Mirza J.I., Haq M.A. (2010). Combining ability analysis of some morpho-physiological traits in Basmati rice. Pak. J. Bot..

[12] Imran M., Masud T., Sakandar H.A. (2014). Studies on physiochemical dimensions of two basmati (Super Basmati, Basmati 385) and two non-basmati (KS-282, IR-6) Pakistani commercial rice varieties. J. Glob. Innov. Agric. Soc. Sci..

[13] Swings J., Van den Mooter M., Vauterin L., Hoste B., Gillis M., Mew T., Kersters K. (1990). Reclassification of the Causal Agents of Bacterial Blight (*Xanthomonas campestris* pv. *oryzae*) and Bacterial Leaf Streak (*Xanthomonas campestris* pv. *oryzicola*) of Rice as Pathovars of *Xanthomonas oryzae* (ex Ishiyama 1922) sp. nov., nom. rev. Int. J. Syst. Evol. Microbiol..

[14] Yasmin S., Hafeez F.Y., Mirza M.S., Rasul M., Arshad H.M., Zubair M., Iqbal M. (2017). Biocontrol of bacterial leaf blight of rice and profiling of secondary metabolites produced by rhizospheric *Pseudomonas aeruginosa* BRp3. Front. Microbiol..

[15] Chukwu S., Rafii M., Ramlee S., Ismail S., Hasan M., Oladosu Y., Magaji U., Akos I., Olalekan K.K. (2019). Bacterial leaf blight resistance in rice: A review of conventional breeding to molecular approach. Mol. Biol. Rep..

[16] Waheed M., Inamullah A.H., Sirajuddin A.H., Khan A., Khan A. (2009). Evaluation of rice genotypes for resistance against bacterial leaf blight. Pak. J. Bot..

[17] Ahsan R., Ullah S., Yaseen I., Fateh F.S., Fayyaz M., Asad S., Jamal A., Sufyan M., Zakria M. (2021). Assessment of bacterial leaf blight incidence and severity in rice growing areas of Pakistan. Pak. J. Agric. Res..

[18] Arif M., Chilvers G., Day S., Naveed S., Woolfe M., Rodionova O.Y., Pomerantsev A., Kracht O., Brodie C., Mihailova A. (2021). Differentiating Pakistani long-grain rice grown inside and outside the accepted Basmati Himalayan geographical region using a ‘one-class’ multi-element chemometric model. Food Control.

[19] Islam W.J. (2017). Management of plant virus diseases; farmer’s knowledge and our suggestions. Host Viruses.

[20] Ali H., Abbasi F.M., Ahmad H. (2016). Bacterial Blight, a serious threat to productivity of rice (*Oryza Sativa* L.), an overview. Int. J. Biosci..

[21] Noman A., Bashir R., Aqeel M., Anwer S., Iftikhar W., Zainab M., Zafar S., Khan S., Islam W., Adnan M. (2016). Success of transgenic cotton (*Gossypium hirsutum* L.): Fiction or reality?. Cogent Food Agric..

[22] Kim S.-M. (2018). Identification of novel recessive gene xa44 (t) conferring resistance to bacterial blight races in rice by QTL linkage analysis using an SNP chip. Theor. Appl. Genet..

[23] Neelam K., Mahajan R., Gupta V., Bhatia D., Gill B.K., Komal R., Lore J.S., Mangat G.S., Singh K. (2020). High-resolution genetic mapping of a novel bacterial blight resistance gene xa-45 (t) identified from *Oryza glaberrima* and transferred to *Oryza sativa*. Theor. Appl. Genet..

[24] Pradhan S., Barik S., Nayak D., Pradhan A., Pandit E., Nayak P., Das S., Pathak H. (2020). Genetics, molecular mechanisms and deployment of bacterial blight resistance genes in rice. Crit. Rev. Plant Sci..

[25] Kim S.-M., Suh J.-P., Qin Y., Noh T.-H., Reinke R.F., Jena K.K. (2015). Identification and fine-mapping of a new resistance gene, Xa40, conferring resistance to bacterial blight races in rice (*Oryza sativa* L.). Theor. Appl. Genet..

[26] Bhasin H., Bhatia D., Raghuvanshi S., Lore J.S., Sahi G.K., Kaur B., Vikal Y., Singh K. (2012). New PCR-based sequence-tagged site marker for bacterial blight resistance gene Xa38 of rice. Mol. Breed..

[27] Kumar P.N., Sujatha K., Laha G., Rao K.S., Mishra B., Viraktamath B., Hari Y., Reddy C., Balachandran S., Ram T. (2012). Identification and fine-mapping of Xa33, a novel gene for resistance to *Xanthomonas oryzae* pv. oryzae. Phytopathology.

[28] Chen X., Liu P., Mei L., He X., Chen L., Liu H., Shen S., Ji Z., Zheng X., Zhang Y. (2021). Xa7, a new executor R gene that confers durable and broad-spectrum resistance to bacterial blight disease in rice. Plant Commun..

[29] Song W.-Y., Pi L.-Y., Wang G.-L., Gardner J., Holsten T., Ronald P.C. (1997). Evolution of the rice Xa21 disease resistance gene family. Plant Cell.

[30] Yang D., Sanchez A., Khush G., Zhu Y., Huang N. (1998). Construction of a BAC contig containing the xa5 locus in rice. Theor. Appl. Genet..

[31] Sun X., Yang Z., Wang S., Zhang Q. (2003). Identification of a 47-kb DNA fragment containing Xa4, a locus for bacterial blight resistance in rice. Theor. Appl. Genet..

[32] Gu K., Yang B., Tian D., Wu L., Wang D., Sreekala C., Yang F., Chu Z., Wang G.-L., White F.F. (2005). R gene expression induced by a type-III effector triggers disease resistance in rice. Nature.

[33] Niño-Liu D.O., Ronald P.C., Bogdanove A.J. (2006). *Xanthomonas oryzae* pathovars: Model pathogens of a model crop. Mol. Plant Pathol..

[34] Cheema K.K., Grewal N.K., Vikal Y., Sharma R., Lore J.S., Das A., Bhatia D., Mahajan R., Gupta V., Bharaj T.S. (2008). A novel bacterial blight resistance gene from *Oryza nivara* mapped to 38 kb region on chromosome 4L and transferred to *Oryza sativa* L.. Genet. Res..

[35] Zhang F., Zhuo D.L., Zhang F., Huang L.Y., Wang W.S., Xu J.L., vera Cruz C., Li Z.K., Zhou Y.L. (2015). Xa39, a novel dominant gene conferring broad-spectrum resistance to *Xanthomonas oryzae* pv. oryzae in rice. Plant Pathol..

[36] Sabar M., Akhter M., Bibi T., Riaz A., Haider Z., Khan A.R., Bibi A. (2019). Basmati rice lines development carrying multiple bacterial blight resistance genes pyramided using the marker-assisted backcross breeding approach. Mol. Breed..

[37] Nayak D., Pandit E., Mohanty S., Barik D., Pradhan S.K. (2015). Marker-assisted selection in back cross progenies for transfer of bacterial leaf blight resistance genes into a popular lowland rice cultivar. ORYZA Int. J. Rice.

[38] Hsu Y.-C., Chiu C.-H., Yap R., Tseng Y.-C., Wu Y.-P. (2020). Pyramiding bacterial blight resistance genes in Tainung82 for broad-spectrum resistance using marker-assisted selection. Int. J. Mol. Sci..

[39] Ullah I., Ali H., Islam M., Ullah W., Haris M., Khan M.Q., Shafiq-ur-Rehman K.K., Khan K., Ghani B. (2020). Molecular analysis of bacterial blight resistance gene XA7 in advance population of rice using STS markers. Int. J. Biosci..

[40] Yugander A., Sundaram R., Ladhalakshmi D., Hajira S., Prakasam V., Prasad M., Sheshu Madhav M., Ravindra Babu V., Laha G.S. (2017). Virulence profiling of *Xanthomonas oryzae* pv. *oryzae* isolates, causing bacterial blight of rice in India. Eur. J. Plant Pathol..

[41] Pradhan S.K., Nayak D.K., Mohanty S., Behera L., Barik S.R., Pandit E., Lenka S., Anandan A. (2015). Pyramiding of three bacterial blight resistance genes for broad-spectrum resistance in deepwater rice variety, Jalmagna. Rice.

[42] Swathi G., Durga Rani C.V., Md J., Madhav M.S., Vanisree S., Anuradha C., Kumar N.R., Kumar N., Kumari K.A., Bhogadhi S.C. (2019). Marker-assisted introgression of the major bacterial blight resistance genes, Xa21 and xa13, and blast resistance gene, Pi54, into the popular rice variety, JGL1798. Mol. Breed..

[43] Suh J.-P., Cho Y.-C., Won Y.-J., Ahn E.-K., Baek M.-K., Kim M.-K., Kim B.-K., Jena K.K. (2015). Development of resistant gene-pyramided japonica rice for multiple biotic stresses using molecular marker-assisted selection. Plant Breed. Biotechnol..

[44] Das G., Rao G.J.N. (2015). Molecular marker assisted gene stacking for biotic and abiotic stress resistance genes in an elite rice cultivar. Front. Plant Sci..

[45] Singh S., Sidhu J., Huang N., Vikal Y., Li Z., Brar D., Dhaliwal H., Khush G.S. (2001). Pyramiding three bacterial blight resistance genes (xa5, xa13 and Xa21) using marker-assisted selection into indica rice cultivar PR106. Theor. Appl. Genet..

[46] Sabar M., Bibi T., Farooq H.U., Haider Z., Naseem I., Mahmood A., Akhter M. (2016). Molecular screening of rice (*Oryza sativa* L.) germplasm for Xa4, xa5 and Xa21 bacterial leaf blight (BLB) resistant genes using linked marker approach. Afr. J. Biotechnol..

[47] Dash A.K., Rao R.N., Rao G., Verma R.L., Katara J.L., Mukherjee A.K., Singh O.N., Bagchi T.B. (2016). Phenotypic and marker-assisted genetic enhancement of parental lines of Rajalaxmi, an elite rice hybrid. Front. Plant Sci..

[48] Eker T., Sari D., Sari H., Tosun H.S., Toker C. (2022). A kabuli chickpea ideotype. Sci. Rep..

[49] Sundaram R., Laha G., Viraktamath B., Sujatha K., Natarajkumar P., Hari Y., Srinivasa Rao K., Reddy C., Balachandran S., Madhav M.S. (2011). Marker assisted breeding for development of bacterial blight resistant rice. J. Plant Pathol..

[50] Dokku P., Das K., Rao G.J.N. (2013). Pyramiding of four resistance genes of bacterial blight in Tapaswini, an elite rice cultivar, through marker-assisted selection. Euphytica.

[51] Pradhan S.K., Pandit E., Pawar S., Baksh S.Y., Mukherjee A.K., Mohanty S.P. (2019). Development of flash-flood tolerant and durable bacterial blight resistant versions of mega rice variety ‘Swarna’ through marker-assisted backcross breeding. Sci. Rep..

[52] Guvvala L.D., Koradi P., Shenoy V., Marella L.S. (2013). Improvement of resistance to bacterial blight through marker assisted backcross breeding and field validation in rice (*Oryza sativa*). Res. J. Biol..

[53] Baliyan N., Malik R., Rani R., Mehta K., Vashisth U., Dhillon S., Boora K.S. (2018). Integrating marker-assisted background analysis with foreground selection for pyramiding bacterial blight resistance genes into Basmati rice. Comptes Rendus Biol..

[54] Suh J.-P., Jeung J.-U., Noh T.-H., Cho Y.-C., Park S.-H., Park H.-S., Shin M.-S., Kim C.-K., Jena K.K. (2013). Development of breeding lines with three pyramided resistance genes that confer broad-spectrum bacterial blight resistance and their molecular analysis in rice. Rice.

[55] Rashid M.M., Nihad S.A.I., Khan M.A.I., Haque A., Ara A., Ferdous T., Hasan M.A.I., Latif M.A. (2021). Pathotype profiling, distribution and virulence analysis of *Xanthomonas oryzae* pv. *oryzae* causing bacterial blight disease of rice in Bangladesh. J. Phytopathol..

[56] Yugander A., Ershad M., Muthuraman P.P., Prakasam V., Ladhalakshmi D., Sheshu Madhav M., Srinivas Prasad M., Sundaram R.M., Laha G.S. (2022). Understanding the variability of rice bacterial blight pathogen, *Xanthomonas oryzae* pv. *oryzae* in Andhra Pradesh, India. J. Basic Microbiol..

[57] Zhang G.-Q., Angeles E., Abenes M., Khush G., Huang N. (1996). RAPD and RFLP mapping of the bacterial blight resistance gene xa-13 in rice. Theor. Appl. Genet..

[58] Sanchez A., Brar D., Huang N., Li Z., Khush G.S. (2000). Sequence tagged site marker-assisted selection for three bacterial blight resistance genes in rice. Crop Sci..

[59] Shanti M., Shenoy V., Devi G.L., Kumar V.M., Premalatha P., Kumar G.N., Shashidhar H., Zehr U., Freeman W.H. (2010). Marker-assisted breeding for resistance to bacterial leaf blight in popular cultivar and parental lines of hybrid rice. J. Plant Pathol..

[60] Bojun M., Wenming W., Bin Z., Yongli Z., Lihuang Z., Wenxue Z. (1999). Studies of PCR marker for the rice bacterial blight resistance gene Xa-4. Yi Chuan Hered..

[61] Yoshimura S., Yoshimura A., Iwata N., McCouch S.R., Abenes M.L., Baraoidan M.R., Mew T.W., Nelson R.J. (1995). Tagging and combining bacterial blight resistance genes in rice using RAPD and RFLP markers. Mol. Breed..

[62] Ronald P.C., Albano B., Tabien R., Abenes L., Wu K.-S., McCouch S., Tanksley S.D. (1992). Genetic and physical analysis of the rice bacterial blight disease resistance locus, Xa21. Mol. Gen. Genet..

[63] Kauffman H.E. (1973). An improved technique for evaluat-ing resistance of rice varieties to *Xanthomonas oryzae*. Plant Dis. Rep..

